# High-performance freestanding supercapacitor electrode based on polypyrrole coated nickel cobalt sulfide nanostructures

**DOI:** 10.1038/s41598-022-08691-2

**Published:** 2022-03-17

**Authors:** Mohammad Barazandeh, Sayed Habib Kazemi

**Affiliations:** Department of Chemistry, Institute for Advanced Studies in Basic Sciences (IASBS), 45137-66731 Zanjan, Iran

**Keywords:** Electrochemistry, Energy, Materials chemistry

## Abstract

In the present work, we report the successful fabrication of dandelion-like Nickel–Cobalt Sulfide@Polypyrrole microspheres through the hydrothermal method and its possible application as a binder-free electrode in supercapacitors. This electrode exhibited low charge transfer resistance with a remarkable specific capacitance of 2554.9 F g^−1^ at 2.54 A g^−1^, in addition to considerable cycle life stability. Also, an asymmetric device was prepared using NiCo_2_S_4_@PPy/NF as positive and rGO/NF as negative electrodes. This asymmetric supercapacitor exhibited a specific capacitance of 98.9 F g^−1^ at 1.84 A g^−1^ and delivered an energy density of 35.17 Wh kg^−1^ at a power density of 1472 W kg^−1^. Such a remarkable performance can be originated from the synergy effect of NiCo_2_S_4_ and PPy and the direct deposition of the composite on the current collector. Our findings suggest the dandelion-like NiCo_2_S_4_@PPy as a promising material for making high-performance supercapacitors.

## Introduction

In recent years, energy storage is becoming one of the most essential problems in the protection of the environment, prompt and stable economic growth^1^. This is becoming more critical due to increase in the global warming problems and rapid depletion of fossil fuels. supercapacitors as novel devices for energy storage have attracted great attention because of their exciting properties such as high power density, short charging times, long life stability, safety, low weight and good reversibility^2^. In General, different types of materials are used in supercapacitors, (1) carbonaceous materials such as activated carbon, graphene and its derivatives, carbon nanotubes, etc., (2) conductive polymers, and (3) transition metal oxides^3–7^.

The first type works based on the mechanism of double-layer capacitance (EDLCs), which utilizes the capacitance arising from charge separation at the interface of electrode and electrolyte. Usually, this class of materials maintains a high surface area^8–10^. A second and third class of materials contains electrochemical faradaic supercapacitors, which show fast and reversible faradaic processes at the surface of the electrode materials^11,12^.

Among these, conducting polymers are widely employed as electrode materials for batteries and electrochemical capacitors because of the combination of the electrical features of metals and the benefits of polymers^13^. Polypyrrole (PPy) can be considered as an important conducting polymer for energy storage purposes because of its substantial conductivity (10–100 Sm^−1^), flexibility, good thermal and practical stability as well as being environmentally stable. Also, its easy synthesis, nontoxicity, relatively low cost, excellent redox behavior, and considerable capacitive current make it suitable for energy storage applications^14–16^. Although PPy has suitable properties, more effort is needed to further improve its capacitive performance because of its poor cycling stability and low rate capability^17–19^. An interesting approach to enhance the strength of PPy is its combination with other electrode materials including carbonaceous materials and transition metal oxides/sulfides. In these composites, metal nanostructures play the role as a skeleton and hold the electrode components together, thus improving the PPy stability^20–24^.

Lately, transition metal sulfides including NiS, CuS, Co_9_S_8_, and NiCo_2_S_4_ have been found to be promising electrode materials for faradaic electrochemical capacitors^25–27^. Amongst ternary Ni-Co sulfides, NiCo_2_S_4_ has attracted increasing attention as a new material in supercapacitors, because of its better electronic conductivity and higher capacity compared to nickel and cobalt sulfide, and oxide^28,29^. Numerous nanostructures of NiCo_2_S_4_ with different morphologies such as nanowires, nanotubes, and nanosheets arrays can be synthesized and deposited or directly grown on the conductive substrates, showing remarkable cycling stability^30–32^. Based on these reports, nanostructured NiCo_2_S_4_ can be considered an excellent candidate to make novel nanomaterials with conducting polymers such as PPy to improve stability and capacitive performance.

In the present work, we introduce a simple and facile process to prepare a dandelion-like NiCo_2_S_4_/PPy nanomaterial for supercapacitor application. In this method, NiCo_2_S_4_@PPy was directly deposited on a nickel foam substrate. The combination of NiCo_2_S_4_ and Polypyrrole, and direct deposition of this material on nickel foam (NF) resulted in the excellent capacitive performance such as high capacitance, good cycle life stability, and significant conductivity. Moreover, an asymmetric device based on NiCo_2_S_4_@PPy/NF and rGO/NF electrodes was assembled. It exhibited a specific capacitance of almost 98.9 F g^−1^ with an energy density of 35.17 Wh kg^−1^ at a power density of 1472 W kg^−1^. These results indicate that NiCo_2_S_4_@PPy/NF is a promising electrode for supercapacitor application.

## Experimental

### Materials and chemicals

In the present work, following materials were used to carry out the experiments: Co(NO_3_)_2_·6H_2_O, Ni(NO_3_)_2_·6H_2_O, Na_2_S·9H_2_O, urea, ammonium persulfate, and pyrrole (purchased from the Sigma-Aldrich company). All chemical reagents were of analytical grade and were used without further purification.

### Synthesis of NiCo_2_O_4_

In a typical experiment, 1.748 g of Co(NO_3_)_2_·6H_2_O (6 mmol), 0.873 g of Ni(NO_3_)_2_·6H_2_O (3 mmol), and 2.162 g of urea (36 mmol, excess) were dissolved in 100 mL of deionized water. The solution was transferred into a Teflon-lined stainless-steel autoclave and heated at 100 °C for 6 h. After cooling down to room temperature, the product (violet precipitate) was filtrated and washed with deionized water thoroughly. Finally, the produced NiCo_2_O_4_ was heat-treated at 250 °C for 2 h.

### Synthesis of polypyrrole

Polypyrrole (PPy) was prepared by the chemical oxidative polymerization method. In this method, the monomer was pyrrole, and ammonium persulfate (APS) was used as the oxidizing agent. First, 1.08 mL of pyrrole was dissolved in 100 mL of 1 M hydrochloric acid (HCl) while stirring the solution and placing it in an ice bath (Solution A). In another container, 0.819 mg of ammonium persulfate was dissolved in 100 mL of 1 M HCl, using ultrasonic (solution B). Prior to production of PPy, solution B was placed in an ice bath. In the next step, solution B was quickly and without stirring added to solution A, and the final solution was kept in an ice bath for 3 h until the completion of the polymerization process. The product (black precipitate) was gathered and washed with deionized water and ethanol rigorously and finally dried at room temperature.

### Synthesis of nanomaterial NiCo_2_S_4_@PPy

To synthesize NiCo_2_S_4_@PPy nanomaterial on the NF substrate, 5 mg of the as-prepared PPy was dispersed in 50 mL of deionized water. Then 15 mg of NiCo_2_O_4_ and 52.3 mg of sodium sulfide monohydrate (Na_2_S.9H_2_O) were added (Solution C). A piece of nickel foam and solution C were transferred into a Teflon-lined stainless steel autoclave and heated at 145 °C for 24 h. After naturally cooling to room temperature, NiCo_2_S_4_@PPy/NF was thoroughly washed with deionized water, and then dried at room temperature.

### Characterization

The morphology of the electrode materials was analyzed by scanning electron microscopy with an energy dispersive X-ray accessory (SEM–EDX, VEGA3 TESCAN), in addition to transmission electron microscopy (TEM, Hitachi 200 kV). Phase analysis was studied by the X-ray diffraction method (XRD, D8-advance, Brucker). The X-ray photoelectron spectroscopy (XPS) experiments were carried out on a VG-Microtech Multilab 3000 instrument. Fourier transform infrared spectroscopy (FTIR) was carried out using a Bruker Vector-22 instrument. Porosimetry investigations were performed by N_2_ adsorption/desorption using a Belsorp-BELMAX. The pore volumes and surface area of the nanomaterials were measured using the Brauner-Emmet-Teller (BET) and Barrett-Joyner-Halenda (BJH) equations. Furthermore, the Raman spectra were studied using an Ar ion CW laser (1064 nm) and a Raman spectrometer (Model: Rigako, Japan).

### Electrochemical measurements

All the electrochemical tests were conducted using an Autolab30 electrochemical workstation (Eco Chemie, the Netherlands) and a Zahner/Zennium (Zahner, Germany) at room temperature. The impedance (EIS) measurements were carried out at the open-circuit potential in 100 kHz to 100 mHz range of frequencies with an AC voltage signal of 10 mV. A conventional three-electrode cell was used for the electrochemical studies of NiCo_2_S_4_@PPy/NF in a 3 M KOH solution. Also, NiCo_2_S_4_@PPy/NF was used as the working electrode, a platinum electrode, and an Ag/AgCl, KCl (sat′d) were employed as the counter and reference electrodes, respectively. Then, active electrode materials were placed on the nickel foam (1 × 1 cm^2^) by hydrothermal method and used as working electrode. An asymmetric supercapacitor (ASC) was designed with NiCo_2_S_4_@PPy/NF as the positive electrode and, rGO coated on nickel foam (rGO/NF) as the negative electrode with a filter paper separator containing 3 M of KOH electrolyte (denoted as NiCo_2_S_4_@PPy/NF//rGO/NF). The electrochemical behaviors of NiCo_2_S_4_@PPy in a three-electrode configuration and the ASC device were investigated by cyclic voltammetry (CV), galvanostatic charge–discharge tests, and electrochemical impedance spectroscopy (EIS).

## Results and discussion

### Characterizations

**Figure 1:**
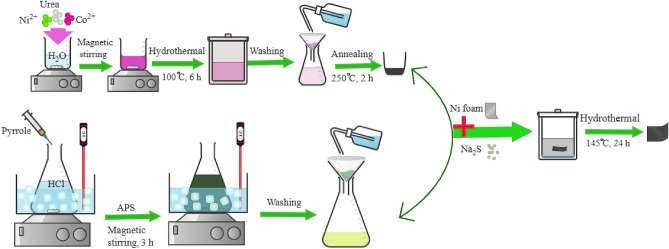
Schematic representation of NiCo_2_S_4_@PPy nanomaterial fabrication.

The fabrication steps of a NiCo_2_S_4_@PPy composite electrode are shown in Fig. 1. In the first step, NiCo_2_S_4_ was prepared via the hydrothermal process which ended with a calcination treatment. Simultaneously, Polypyrrole (PPy) was prepared by the chemical oxidative polymerization method. Then, the NiCo_2_S_4_@PPy nanomaterial was deposited on the Ni foam substrate through a facile hydrothermal method.

**Figure 2:**
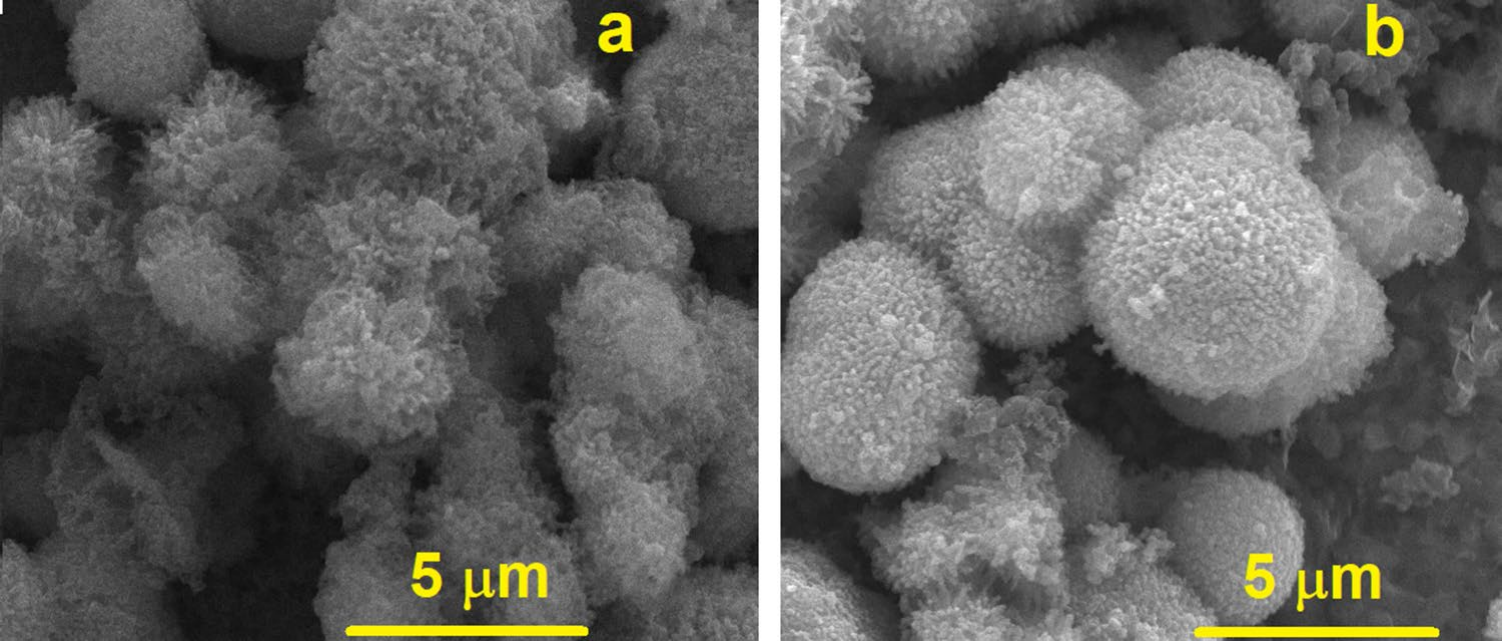
SEM images of NiCo_2_S_4_ (**a**) and NiCo_2_S_4_@PPy nanomaterial (**b**).

**Figure 3:**
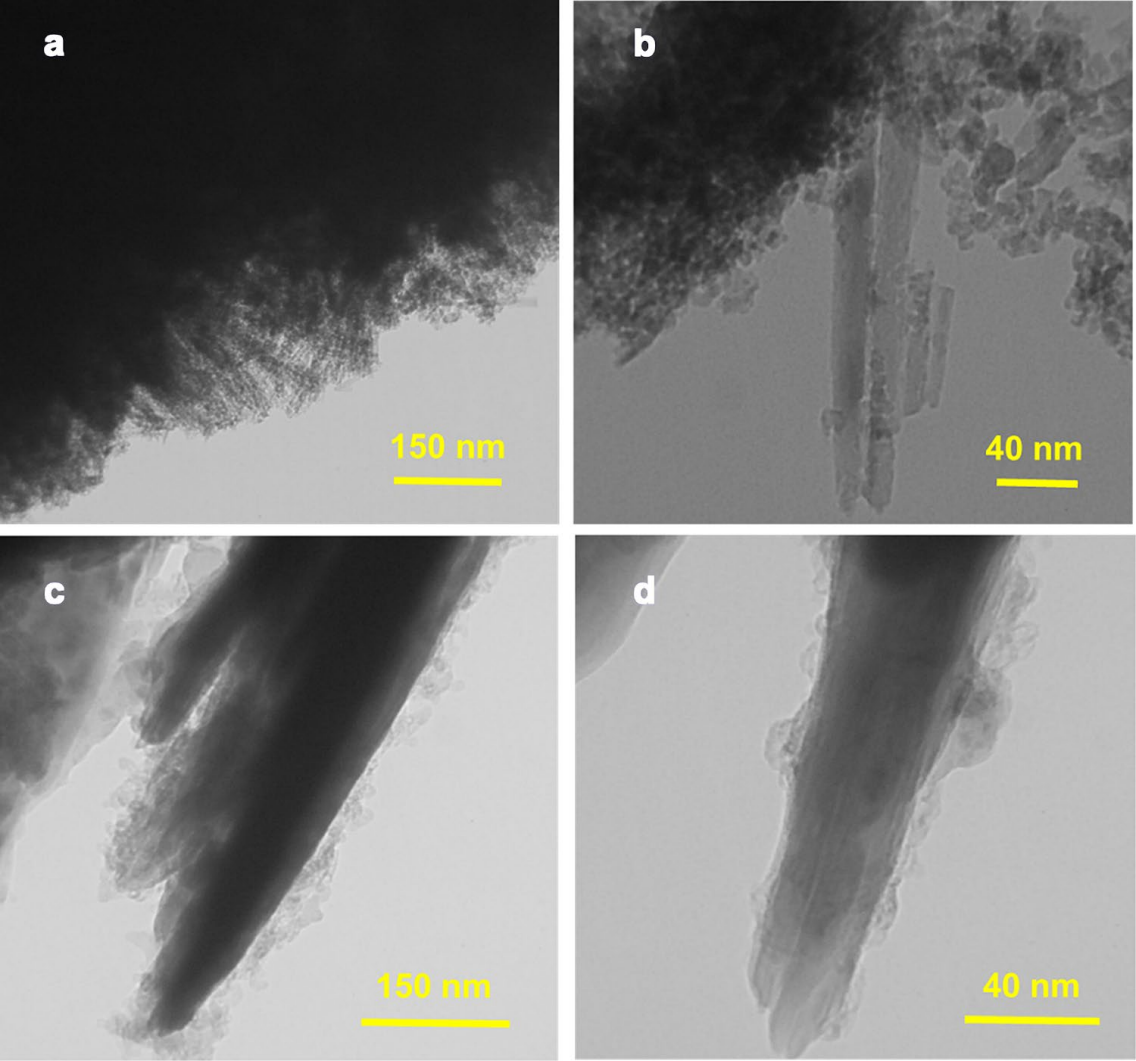
TEM images of (**a**,**b**) NiCo_2_S_4_ and (**c**,**d**) NiCo_2_S_4_@PPy nanomaterials.

The morphology of the prepared NiCo_2_S_4_ and NiCo_2_S_4_@PPy were probed using the SEM and TEM techniques, and the corresponding results are shown in Figs. 2 and 3. As seen, the SEM image of NiCo_2_S_4_ (Fig. 2a) reveals that the NiCo_2_S_4_ microspheres uniformly covered the NF surface. Also, this image shows that the NiCo_2_S_4_ flower-like microsphere actually appears in a porous structure with empty spaces between its nanowires and nanoflakes. This structure can facilitate the diffusion of the electrolyte ions to the deeper active sites of the NiCo_2_S_4_ nanomaterial and the increased real surface area.

Also, Fig. 2b depicts a typical SEM image of the NiCo_2_S_4_@PPy nanomaterial. As seen in Fig. 2b, the original morphology of NiCo_2_S_4_ is maintained after the formation of NiCo_2_S_4_@PPy on the NF surface. As seen in Figs. 2a and 3b, the average radius of the microsphere is smaller than 4 µm in NiCo_2_S_4_ and is increased to almost 5 µm in the NiCo_2_S_4_@PPy nanomaterial. Additionally, it can be concluded that the highly accessible surface of NiCo_2_S_4_@PPy enhances the penetration of the electrolyte ions into the electrode material. In other words, increased accessible surface area and ease of ion and electron diffusion in the porous structure can result in the improved capacitive performance of NiCo_2_S_4_@PPy.

Shown in Fig. 3 are TEM images recorded to check the structural and morphological characteristics of NiCo_2_S_4_ and NiCo_2_S_4_@PPy. Figure 3a represents the hierarchical structure of NiCo_2_S_4_ appearing as many nanowires with an average thickness of almost 15 nm. Also, a higher resolution TEM image of the NiCo_2_S_4_ nanowires is provided in Fig. 3b. It is obvious that the nanowire structure is relatively crystalline and its length may be longer than 100 nm. This is in excellent agreement with the SEM results in which NiCo_2_S_4_ nanospheres consist of many integrated nanowires (Fig. 2a). During the synthesis of the NiCo_2_S_4_@PPy nanomaterial, it is expected to see the enlargement of the radius of the NiCo_2_S_4_ nanowires when they are wrapped with a shell layer of PPy. Figure 3c,d illustrate the NiCo_2_S_4_@PPy nanowires with an amorphous shell of polypyrrole in which the average thickness of the nanowires is almost 20 nm (Fig. 3d). It is noteworthy that the TEM images of NiCo_2_S_4_@PPy support the SEM observations indicate the enlargement of the nanowires when they are covered by PPy.

**Figure 4:**
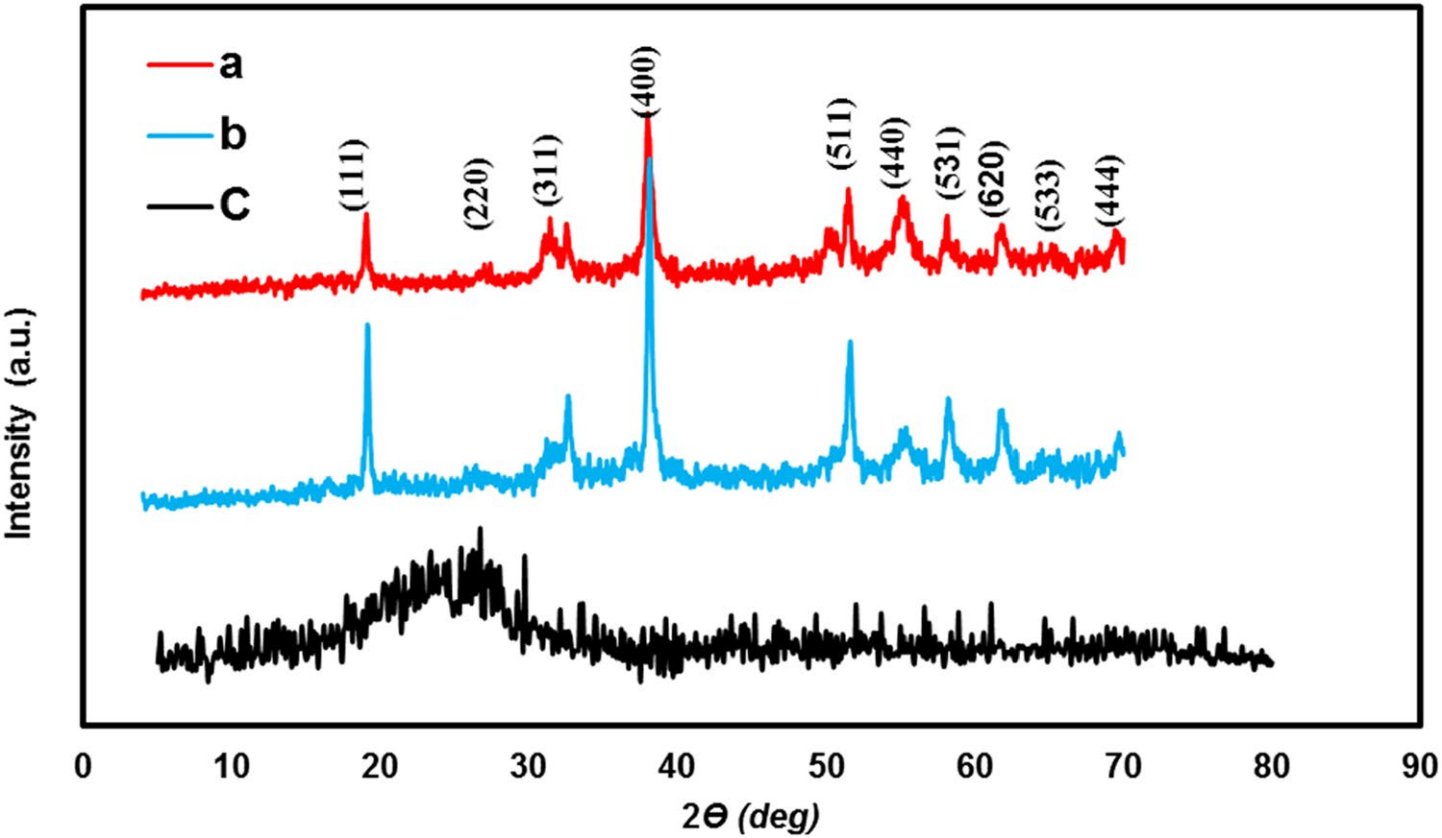
(**a**) XRD patterns of NiCo_2_S_4_, (**b**) NiCo_2_S_4_@PPy and, (**c**) PPy.

The X-ray diffraction technique was employed to study the structure of NiCo_2_S_4_ and NiCo_2_S_4_@PPy and their corresponding results are shown in Fig. 4. As seen in Fig. 4a, a typical XRD plot of NiCo_2_S_4_ is displayed in which the diffraction peaks located at 16.5^°^, 26.8^∘^, 31.5^∘^, 38.1^∘^, 50.4^∘^, 55.2^∘^, 58.4̊, 62.5^∘^, 65^∘^ and, 68.5^∘^ can be assigned to the respective (111), (220), (311), (400), (511), (440),(531), (620), (533) and (444) planes of the cubic phase of NiCo_2_S_4_ (JCPDS 20-0782)^1,33–35^. Also, the NiCo_2_S_4_@PPy composite (Fig. 4b) displays a similar XRD pattern to that of nickel–cobalt sulfide. This is probably due to the thin layer of the amorphous structure of PPy (Fig. 4c) which does not yield significant characteristic reflection peaks in the recorded pattern of NiCo_2_S_4_@PPy^36^. Thermal gravimetric analysis (TGA) was employed to check the thermal stability of the pure PPy. Figure Sx depicts the TGA curve of pure PPy. Also, the TGA results display three stages of weight loss in the mentioned range. The evaporation of water molecules from the polymer structure is responsible for the first step of weight loss (5.97 percent) up to 150 °C. At 250 °C, the sample loses a significant amount of weight because of the initialization of the PPy structure breakdown. In the third step of weight loss, from 250 to 900 °C, the PPy backbone decomposes completely^37–39^.

**Figure 5:**
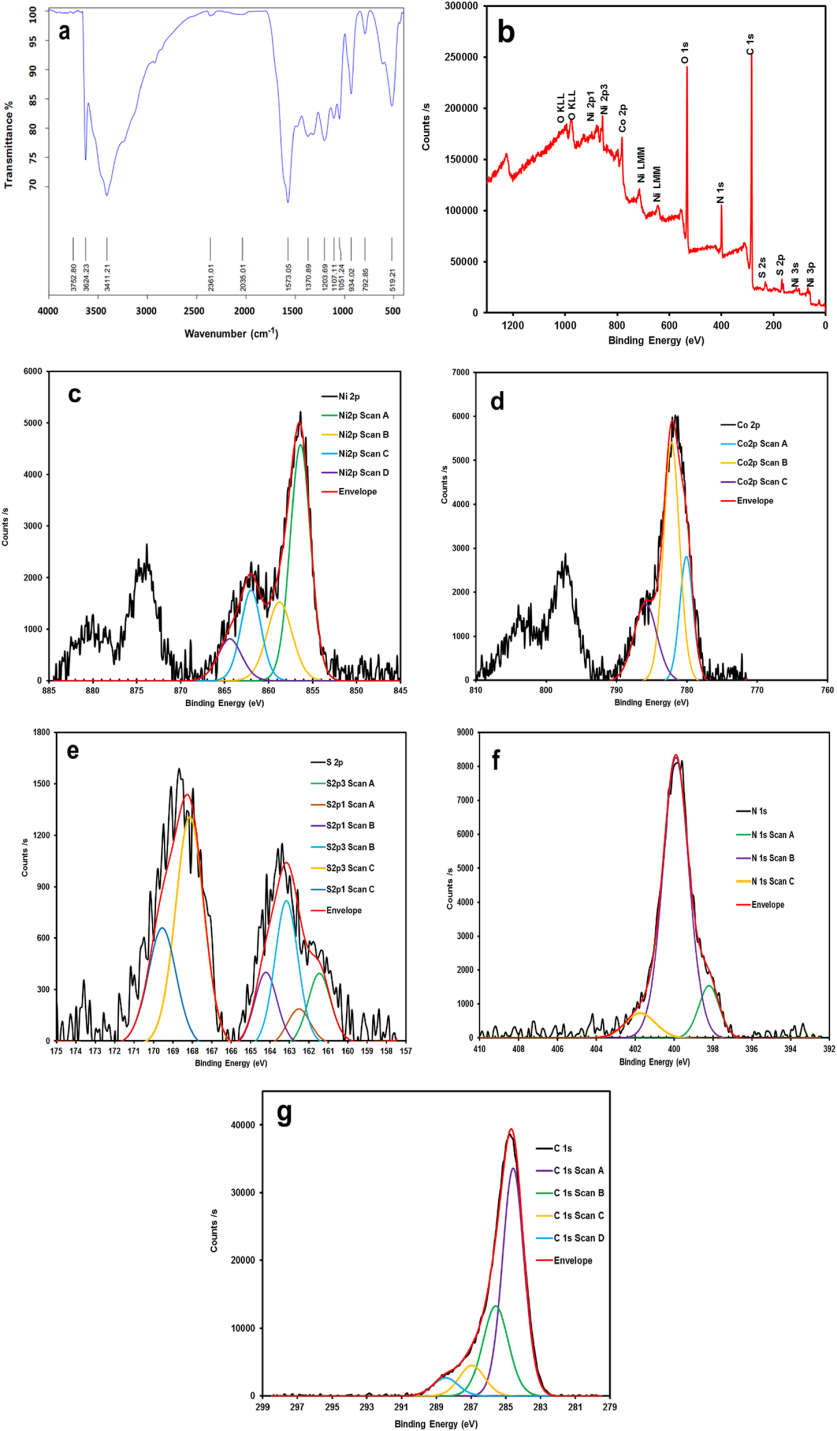
(**a**) FT-IR spectrum, (**b**) XPS survey, and (**c**–**g**) XPS spectra of Ni 2p, Co 2p, S 2p, N 1 s, and C 1 s for the NiCo_2_S_4_@PPy nanomaterial, respectively.

Moreover, to check the functional groups of the synthesized nanostructure, FT-IR analysis was performed and the corresponding FT-IR spectrum is presented in Fig. 5a. As seen in this figure, the peaks appear at 519.21, 792.85 cm^−1^ (symmetrical stretching) and 1107 cm^−1^ (asymmetrical stretching) are attributed to the Ni–S or Co–S vibrations of NiCo_2_S_4_^40^. The peak at 934.02 cm^−1^ could be attributed to the C-H deformation vibration in the –CH=CH − group^41^. In addition, the broad band located at 3411 cm^−1^ can be assigned to the N–H stretching vibration in the pyrrole rings^42^. To further investigate the nanomaterial structure, Raman analysis was performed on the NiCo_2_S_4_@PPy samples (Fig. S1). The Raman spectrum recorded for pure PPy shows two peaks at 1552 cm^−1^ and 1340 cm^−1^. These peaks can be assigned to the p-conjugated structure and the stretching mode of the ring of the polymer, respectively. Also, a peak appears at 1037 cm^−1^ which may be attributed to the C–H in-plane deformation. The Raman spectrum of NiCo_2_S_4_@PPy shows clear peaks of PPy, which can be observed at 1552, 1340 and 1037 cm^−1^. Additionally, the peaks that appear at 516.3 and 668 cm^−1^ are the characteristic peaks for the F2g and A1g modes of NiCo_2_S_4_^43,44^.

X-ray photoelectron spectroscopy (XPS) studies were implemented to obtain a more detailed elemental analysis and the chemical state of the NiCo_2_S_4_@PPy nanomaterial. The survey spectrum of the NiCo_2_S_4_@PPy nanomaterial (Fig. 5b) demonstrates the presence of C, N, S, O, Ni, and Co. Figure 5c–g represent the typical fitted Ni 2p, Co 2p, S 2p, N 2p and, C 1 s peaks of NiCo_2_S_4_@PPy. The XPS spectrum of Ni 2p and Co 2p can be fitted with two spin-orbits and two satellites (shake-up, shows as “sat”). As seen in Fig. 5c, the fitting peaks at 856.39 eV and 873.58 eV can be indexed to Ni 2p_3/2_ and Ni 2p_1/2_, respectively^45^. Furthermore, Fig. 5d demonstrates the Co 2p XPS spectrum. The fitting peaks corresponding to 780.7 and 796.2 eV are correlated to Co 2p_3/2_ and Co 2p_1/2_, respectively^46,47^. Also, the S 2p XPS spectrum is shown in Fig. 5e. The peaks that appear at 161.5 eV and 163.4 eV are matched to S 2P_1/2_ and S 2p_3/2_, respectively^48,49^. Based on the XPS analysis, the chemical composition of NiCo_2_S_4_@PPy contains Ni^2+^, Ni^3+^, Co^2+^, Co^3+^ and, S^2−^, confirming the previously reported results^33,50^. The N 1 s XPS plot can be deconvoluted into three components, as displayed in Fig. 5f. The fitting peak at the binding energies of 398.2 eV, 399.9 eV, and, 401.7 eV correspond to imine-like (–C=N–), pyrrole (–NH–), and positively charged nitrogens (–NH^+^–), respectively^48,51,52^. Additionally, for the C 1 s XPS spectrum presented in Fig. 5g, the fitting peaks appear at the binding energies of 284.57, 285.59, 286.97, and 288.47 eV may be attributed to the β-C, α-C, C=N, and –C=N bonds, individually^53^.

### Electrochemical studies

**Figure 6:**
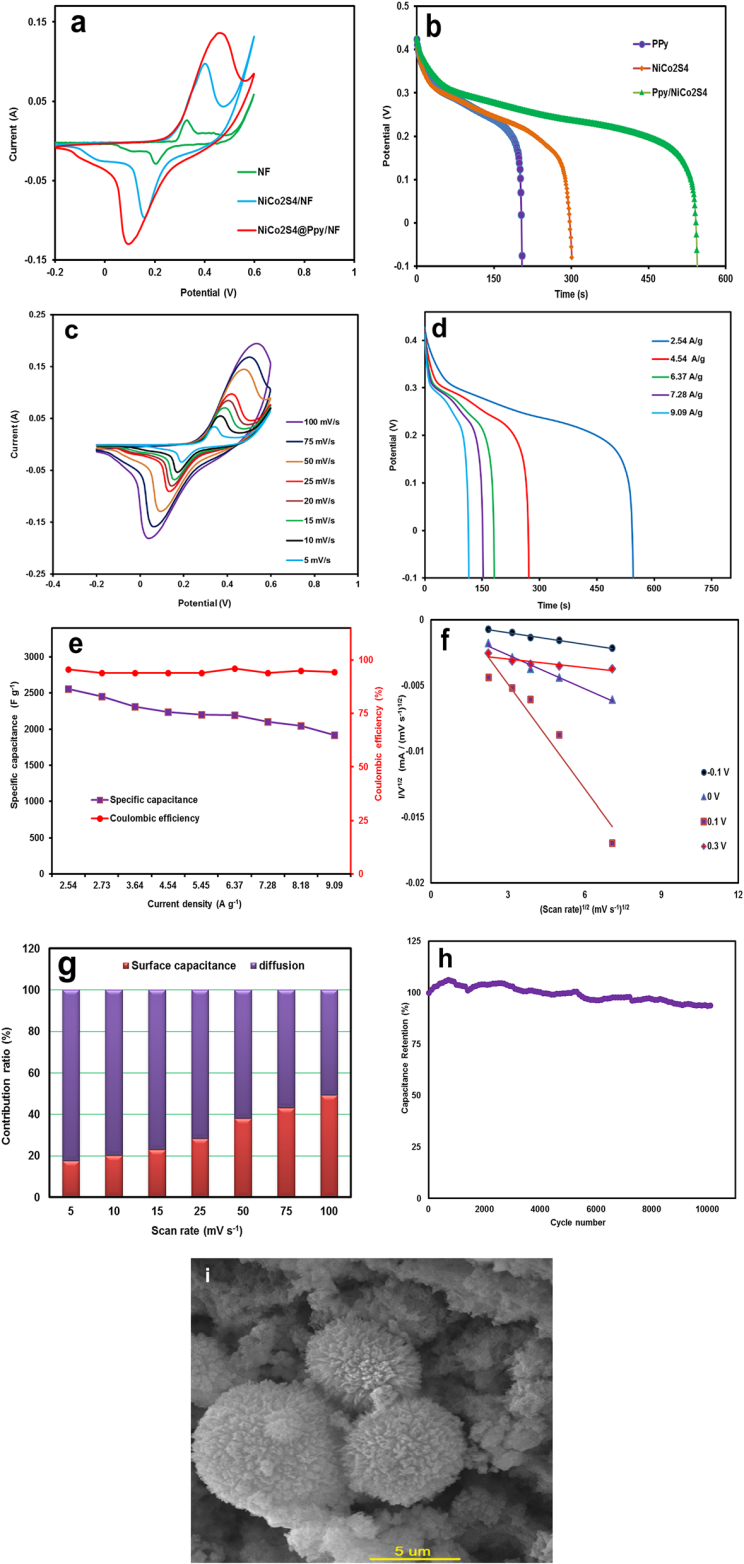
(**a**) Voltammograms recorded for the Ni-foam, NiCo_2_S_4_, and NiCo_2_S_4_@PPy nanomaterials at a scan rate of 50 mV s^−1^, (**b**) comparison of the GCD curves of NiCo_2_S_4_@PPy, NiCo_2_S_4_, and Ni foam at a current density of 2.54 A g^−1^, (**c**) CV curves of NiCo_2_S_4_@PPy electrode at different scan rates, (**d**) GCD curves of the NiCo_2_S_4_@PPy electrode at different current densities, (**e**) plots of specific capacitance and Coulombic efficiency against current density, (**f**) The plots of i(v)/*v*^1/2^ versus *v*^1/2^ at various potentials, (**g**) Surface capacitive and diffusion-controlled contribution to the capacitance at various scan rates, (**h**) cycling life test of the NiCo_2_S_4_@PPy electrode at a scan rate of 100 mV s^−1^, and (**i**) the SEM image of NiCo_2_S_4_@PPy electrode at a scan rate of 100 mV s^−1^ after 2100 cycles.

To investigate the electrochemical behavior of the NiCo_2_S_4_@PPy nanomaterial, cyclic voltammetry, galvanostatic charge/discharge and, electrochemical impedance spectroscopy were carried out in a three-electrode cell in an alkaline solution of 3 M of KOH. Also, NiCo_2_S_4_@ PPy/NF, Pt and an Ag/AgCl, KCl (sat′d) were used as working, counter and reference electrodes, respectively. Figure 6a exhibits a comparison of the voltammograms for bare NF, NiCo_2_S_4_/NF, and NiCo_2_S_4_@PPy/NF electrodes at a scan rate of 50 mV s^−1^ within a potential range of -0.2 to 0.6 V. As seen in the Fig. 6a, a pair of redox peaks was appeared for NiCo_2_S_4_/NF and NiCo_2_S_4_@PPy/NF electrodes due to the Faradaic charge storage mechanism for the electrodes. These peaks may be attributed to the reversible redox processes of Ni^2+^/Ni^3+^ and Co^2+^/Co^3+^/Co^4+^ as follows:

1 $${\text{NiCo}}_{2} {\text{S}}_{4} + {\text{OH}}^{ - } + {\text{H}}_{2} {\text{O}} \rightleftharpoons {\text{NiSOH}} + 2{\text{CoSOH}} + 2{\text{e}}^{ - }$$

2 $${\text{CoSOH}} + {\text{OH}}^{ - } \rightleftharpoons {\text{CoSO}} + {\text{H}}_{2} {\text{O}} + {\text{e}}^{ - }$$

As expected, the NiCo_2_S_4_@PPy/NF electrode exhibits much higher specific capacitance than both NiCo_2_S_4_/NF and bare NF electrodes. This fact was also confirmed by GCD measurements (Fig. 6b). The obvious deviation from linear discharge that appears as a plateau, further confirms the Faradaic behavior of the electrode materials. Moreover, the longer discharge time approves the higher specific capacitance for the NiCo_2_S_4_@PPy/NF electrode. This is in a good agreement with voltammetry results. Figure 6c depicts the typical cyclic voltammetry curves of the NiCo_2_S_4_@PPy/NF electrode at various scan rates ranging from 5 up to 100 mV s^−1^ within a potential range of − 0.2 to 0.6 V (vs. Ag/AgCl). The shape of the CV curves was almost unchanged even at the high scan rate of 100 mV s^−1^, indicating the remarkable capacitive behavior and high-rate capability of the NiCo_2_S_4_@PPy electrode material. The galvanostatic charge/discharge tests were correspondingly performed at various current densities ranging from 2.54 to 9.09 A g^−1^ in a potential range of − 0.1 to 0.45 V (Fig. 6d). At a current density of 2.54 A g^−1^, the specific capacitance of the NiCo_2_S_4_ PPy/NF electrode was estimated to be 2554.9 F g^−1^. The specific capacitance and rate capability at various discharge currents are presented in Fig. 6e and Table S1. It is clear that the specific capacitance was slightly decreased as the current density increased from 2.54 to 9.09 A g^−1^. Moreover, the NiCo_2_S_4_@PPy/NF electrode still maintained 72% of its maximum specific capacitance at a current of 9.09 A g^−1^. The volumetric capacitance was also estimated, and the matching curve is shown in Fig. S3. A volumetric capacitances of 1847.18 F cm^−3^ at a current of 2.54 A g^−1^, and 1387.57 F cm^−3^ at a current of 9.09 A g^−1^ were achieved. These findings verify that the NiCo_2_S_4_ PPy/NF electrode can be considered as an excellent alternative electrode to use in high-performance supercapacitors. The diffusion controlled and surface capacitive charge storage mechanisms contribute to the total capacitance of NiCo_2_S_4_@PPy/NF. The current dependency on the scan rate, ν, is determined by the following equation in theory^29^:

$$i={av}^{b}$$

where ‘*a*’ and ‘*b*’ are adjustable parameters, *i* is the current and *v* is the scan rate. When *b* = 1, the surface capacitive process is the dominant mechanism of charge storage; otherwise, when *b* = 0.5, diffusion-limited processes govern the current density (battery-type behavior). The *b*-values of NiCo_2_S_4_@PPy were calculated within the potential window of the observed voltammograms. The *b*-values near 1.0 within the potential range of − 0.1 and 0.1 V, indicate the pure capacitive charge storage mechanism controlling the current. However, the *b*-values of 0.65–0.7 were obtained for the potential range of 0.3–0.4 V, implying the contribution of diffusion-controlled and surface capacitive processes. It is noteworthy that NiCo_2_S_4_@PPy/NF exhibits a diffusion-controlled (battery-type) feature, in addition to capacitive charge storage. Thus, the peak current can be considered as the sum of the currents for capacitive processes (k_1_v) and diffusion-limited processes (k_2_v^1/2^), according to the following equation:

$$i\left(V\right)={k}_{1}v+ {k}_{2}{v}^{1/2}$$

The slope and intercept of the lines of ${v}^{1/2}$ against $i(V)/{v}^{1/2}$ can be used to compute k_1_ and k_2_ (Fig. 6f). The contribution fraction of each capacitance mechanism at different scan rates is displayed in Fig. 6g. It can be concluded that the diffusion-controlled process is the major source of charge storage behavior in NiCo_2_S_4_@PPy/NF. Additionally, the contribution of the capacitive charge storage is increased by increasing the scan rates because of the limited time for ion transport into deeper sites. Therefore, at the higher scan rates, the contribution of the redox processes will decrease. The practical cycle-life stability of the NiCo_2_S_4_@PPy/NF electrode was investigated for 10,000 successive cycles. As shown in Fig. 6h, a remarkable specific capacitance retention of almost 92% after 10,000 cycles demonstrates considerable cycle-life stability. To further check the morphology changes during the long-term cycle stability test, SEM analysis was carried out for the NiCo_2_S_4_@PPy/NF electrode after 2100 cycles. It is obvious that the primary structure is almost well-maintained (shown in Fig. 6i). As a result, positive cooperation of PPy and NiCo_2_S_4_ results in the structural integrity of the NiCo_2_S_4_@PPy nanomaterial and improves the cycling performance and stability.

**Figure 7:**
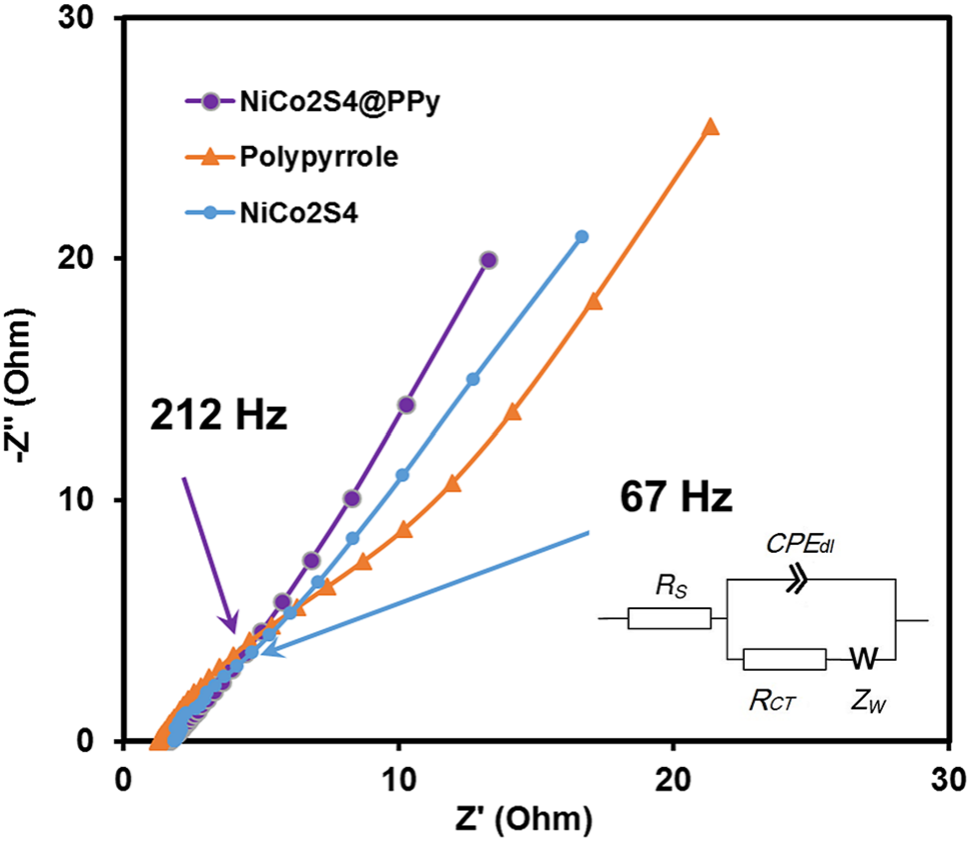
EIS results for the PPy, NiCo_2_S_4_, and NiCo_2_S_4_@PPy electrodes: knee point frequencies for NiCo_2_S_4_ and NiCo_2_S_4_@PPy electrodes are emphasized the corresponding arrows. The inset depicts a modified Randles electrical circuit used to fit the EIS results.

Electrochemical impedance spectroscopy tests were further conducted to inspect the electrical conductivity and ion diffusion properties of the PPy/NF, NiCo_2_S_4_/NF, and NiCo_2_S_4_@PPy/NF electrodes. Figure 7 depicts the Nyquist plots of the electrodes at open circuit potential (OCP). The expanded high-frequency region of the Nyquist plot is shown as inset in Fig. 7, and demonstrates that the NiCo_2_S_4_@PPy/NF electrode has lower R_ct_ compared to the PPy/NF and NiCo_2_S_4_/NF electrodes. Also, at the low-frequency region of these Nyquist plots of the NiCo_2_S_4_@PPy/NF and NiCo_2_S_4_/NF electrodes, diffusion impedance appears. It is an evidence for a substantial capacitive behavior of the electrode materials. To numerically compare the electrochemical impedance parameters of the different electrodes, we have used a modified Randles equivalent circuit to fit the experimental EIS results (shown as an inset in Fig. 7). Also, details of the fitting step are shown in Table S4 (supporting information). As can be seen, both Rs and Rct are smaller for NiCo_2_S_4_@PPy/NF compared with the PPy/NF and NiCo_2_S_4_/NF electrodes (1.12 and 1.64 ohms for NiCo_2_S_4_@PPy/NF reaching 1.25 and 12.37 ohms for pure PPy/NF and 1.84 and 5.32 ohms for the NiCo_2_S_4_/NF counterparts, respectively). These findings confirm the substantial interaction between the NiCo_2_S_4_ and PPy constituents of the electrode and the robust contact with NF substrate. Such an excellent interaction provides suitable pathways for electrons and ions in the dandelion-like structure of NiCo_2_S_4_, a remarkable electrical contact, and fast redox reactions of the nanomaterial.

It is noticeable that the studied nanomaterials show almost pure capacitive behavior at even high frequencies of 67 and 212 Hz for the NiCo_2_S_4_/NF and NiCo_2_S_4_@PPy/NF electrodes, respectively. The NiCo_2_S_4_@PPy nanomaterial shows superior capacitive behavior compared to the NiCo_2_S_4_/NF, supporting the electrochemical results of cyclic voltammetry and charge/discharge tests^54^. Moreover, the NiCo_2_S_4_@PPy electrode material preserves a significantly shorter relaxation time constant compared to the NiCo_2_S_4_ or PPy electrodes. This behavior stems from the substantial electrical conductivity of the nanomaterial electrode due to the notable synergy between NiCo_2_S_4_ and PPy in the nanomaterial and enhanced electrical contact between the NiCo_2_S_4_@PPy and NF support without using any insulating polymers as binder.

**Figure 8:**
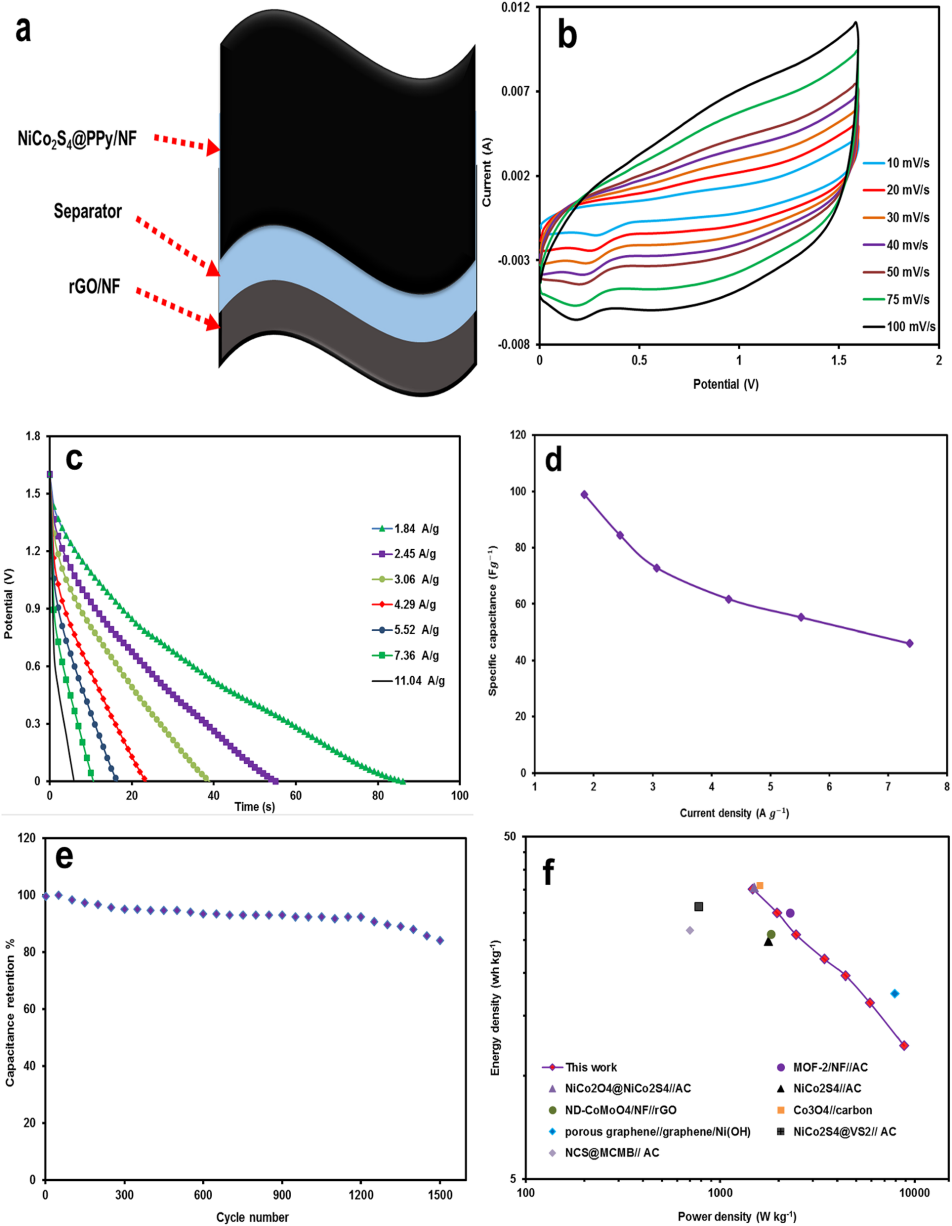
(**a**) Schematic demonstration of the ASC device, (**b**) CV curves of NiCo_2_S_4_@ PPy//rGO at different scan rates, (**c**) GCD curves of recorded against current density (1.84–11.04 A g^−1^), (**d**) specific capacitance against the current densities, (**e**) long-life cycling test at a scan rate of 150 mV s^−1^ for 1500 cycles, and (**f**) Ragone plot of the NiCo_2_S_4_@PPy//rGO/NF ASC device.

To further estimate the practical application of NiCo_2_S_4_@PPy/NF, an asymmetric supercapacitor (Fig. 8a) was constructed. Then, the reduced graphene oxide was brushed on a piece of NF (rGO/NF) as the negative electrode, and coupled to NiCo_2_S_4_@PPy/NF as the positive electrode (device notation: NiCo_2_S_4_@PPy//rGO/NF). The NiCo_2_S_4_@PPy//rGO/NF electrode properties of were examined using cyclic voltammetry and galvanostatic charge/discharge tests. Figure 8b displays typical voltammograms of the ASC device in a voltage span of 0 to 1.6 V at a wide range of scan rates (10–100 mV s^−1^). It is obvious that cyclic voltammograms almost keep their quasi-rectangular shape with the increasing scan rate, confirming the perfect capacitive performance and rapid current response of the asymmetric supercapcitor. Figure 8c exhibits the discharge branch of the GCD curves at various currents from 1.84 to 11.04 A g^−1^, in a voltage range of 0–1.6 V. The specific capacitance of the ASC device was calculated according to the corresponding GCD cruves. A maximum specific capacitance of 98.92 F g^−1^ was achieved at a current density of 1.84 A g^−1^ for the NiCo_2_S_4_@PPy//rGO/NF ASC device. It is notable that the ASC device retained almost 55% of its initial capacitance, when the current increased from 1.84 to 6 A g^−1^ (Fig. 8d). These results reveal the remarkable rate capability of the NiCo_2_S_4_@PPy//rGO ASC device. The long-term cycle stability as a critical parameter for the real application of a supercapacitor was inspected by voltammetry measurements at the high scan rate of 150 mV s^−1^ for 1500 cycles. As shown in Fig. 8e, the NiCo_2_S_4_@PPy//rGO/NF device maintains 85% of its primary capacitance after approximately 1500 cycles. Additionally, power and energy densities are the essential parameters to investigate the electrochemical performance:

3 $$P=\frac{E}{t}$$

4 $${\text{E}}=\frac{1}{2}{\text{C}}{V}^{2}$$

Figure 8f illustrates the Ragone plot (energy against power density) of the asymmetric supercapacitor (ASC). The NiCo_2_S_4_@PPy/NF//rGO/NF exhibits a maximum energy density of 35.17 Wh kg^−1^ at a power density of 1472.29 W kg^−1^. When the power density increases to 8832 W kg^−1^, the ASC device still retains the energy density of 12.26 Wh kg^−1^. These values are comparable to the recently reported similar works, such as NiCo_2_S_4_@VS_2_//AC (31.2 Wh kg^−1^ at 775 W kg^−1^), NiCo_2_O_4_@ NiCo_2_S_4_//AC (35.6 Wh kg^−1^ at 1500 W kg^−1^), NiCo_2_S_4_//AC (24.78 Wh kg^−1^ at 1770.1 W kg^−1^), GRH-NiCo_2_S_4_//YP-50 (19 Wh kg^−1^ at 703 W kg^−1^), NiCo_2_S_4_@Ni(OH)_2_@PPy//AC (34.67 Wh kg^−1^ at 120.13 W kg^−1^), NiCo_2_S_4_@PPy-50//AC (34.62 Wh kg^−1^ at 120.19 W kg^−1^), ND-CoMoO_4_/NF//rGo (26 Wh kg^−1^ at 1821 W kg^−1^), MOF-2/NF//AC (30 Wh kg^−1^ at 2285.7 W kg^−1^), NCS@MCMB//AC (26.6 Wh kg^−1^ at 700 W kg^−1^), RGO/PPy/(Ni-Co)LDH//RGO (41.9 Wh kg^−1^ at 698 W kg^−1^), Co_3_O_4_//carbon (36 Wh kg^−1^ at 1600 W kg^−1^), and Porous Graphene//Graphene/Ni(OH) (17.4 Wh kg^−1^ at 7900 W kg^−1^)^34,43,55–64^. Moreover, Table S2 shows the electrochemical characteristics of the present work compared to the previously reported works. Thus, the NiCo_2_S_4_ PPy/NF electrode shows a superior electrochemical performance. Such excellent behavior can originate from the more accessible electroactive spots for the redox reactions and porous structure, which enhance the electron transfer and ion diffusion during the charge/discharge processes. These results reveal that the NiCo_2_S_4_@PPy electrode material has a high electrochemical performance, and it is a promising electrode for supercapacitor applications.

## Conclusion

In summary, the NiCo_2_S_4_@PPy/NF electrode was successfully fabricated by a facile hydrothermal method. This nanomaterial exhibited an exceptional specific capacitance of 2554.88 Fg^−1^ at 2.54 A g^−1^ discharge current, a remarkable long-term cycling stability (92% of capacitance retention even after 10,000 cycles), and low charge-transfer resistance. An asymmetric supercapacitor based on the NiCo_2_S_4_@PPy/NF as the positive electrode and rGO@NF as the negative electrode was assembled. The as-prepared ASC device can produce a great specific capacitance of almost 98.9 F g^−1^ at a discharge current of 1.84 A g^−1^ in a potential range of 0.0 to + 1.6 V. Also, this asymmetric device exhibits notable cycle life stability of 85% after 1500 cycles. Therefore, the NiCo_2_S_4_@PPy/NF electrode with excellent electrochemical performance, is a suitable candidate to be used in supercapacitors.

## Supplementary Information

Supplementary Information.
