# Author Correction: High-performance freestanding supercapacitor electrode based on polypyrrole coated nickel cobalt sulfide nanostructures

**DOI:** 10.1038/s41598-023-36722-z

**Published:** 2023-06-15

**Authors:** Mohammad Barazandeh, Sayed Habib Kazemi

**Affiliations:** Department of Chemistry, Institute for Advanced Studies in Basic Sciences (IASBS), 45137‑66731 Zanjan, Iran

Correction to: *Scientific Reports* https://doi.org/10.1038/s41598-022-08691-2, published online 17 March 2022

The original published version of this article contained errors in Figure 2. Both panels showed the NiCo_2_S_4_@PPy nanomaterial and, in addition, Figure 2b partially overlapped with Figure 6i. The error happened during the assembly of the original Figure. The original Figure 2 and accompanying legend appear below.

**Figure 2:**
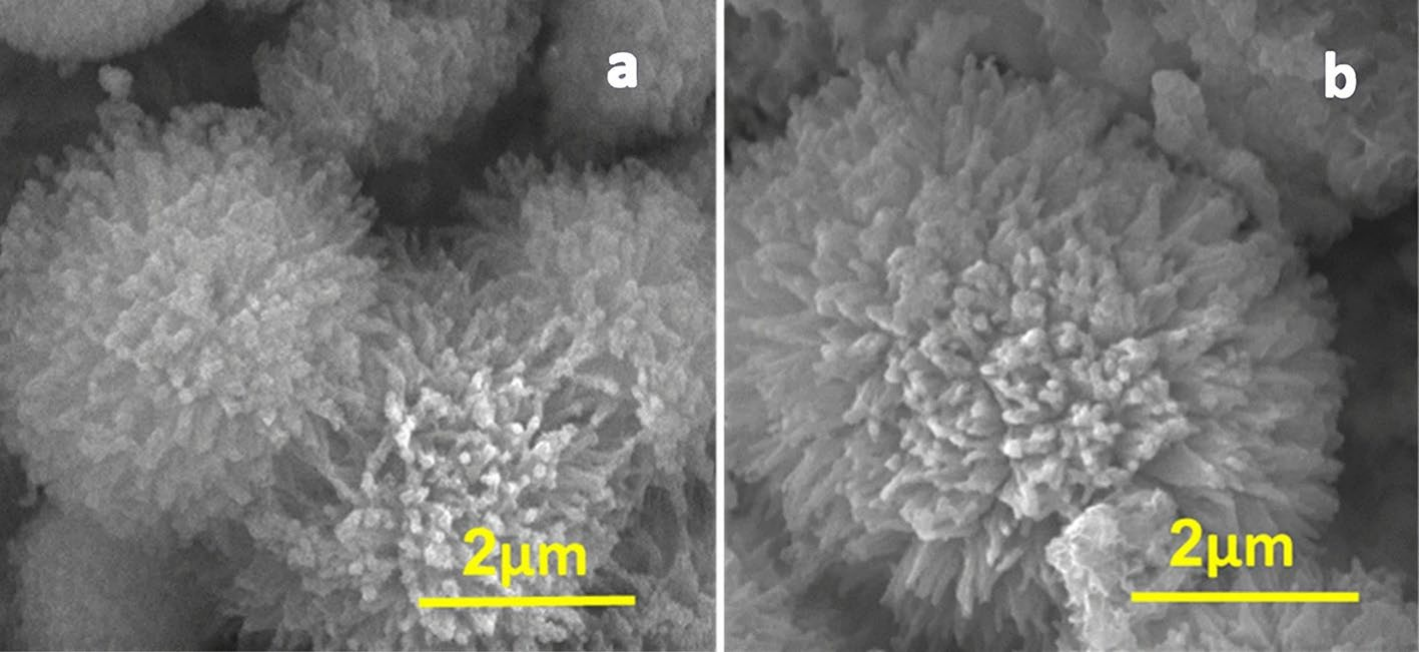
SEM images of NiCo_2_S_4_ (**a**) and NiCo_2_S_4_@PPy nanomaterial (**b**).

The original article has been updated.

