# Supplementary material for: High-performance freestanding supercapacitor electrode based on polypyrrole coated nickel cobalt sulfide nanostructures
Source: Sci Rep. 2022 Mar 17;12:4628. doi: 10.1038/s41598-022-08691-2 (PMC8930993; doi:10.1038/s41598-022-08691-2)
Supplement: Supplementary file 1 — Supplementary Information. [file 41598_2022_8691_MOESM1_ESM.pdf]

Supporting Information

High-Performance Freestanding Supercapacitor Electrode Based on Polypyrrole Coated Nickel Cobalt Sulfide Nanostructures

Mohammad Barazandeh, Sayed Habib Kazemi*

Department of Chemistry, Institute for Advanced Studies in Basic Sciences (IASBS), Zanjan 45137-66731, Iran

* Corresponding Author: Sayed Habib Kazemi (habibkazemi@iasbs.ac.ir)

Table S-1. Galvanostatic charge-discharge results used to estimate the Coulombic efficiency

Current density (A g^{-1})	Charging time (s)	Discharge time (s)	Coulombic efficiency (%)
2.54	571	542	96
3.64	367	343	94
4.54	284	266	94
5.45	231	219	95
6.37	194	186	96
7.28	165	156	94
8.18	142	135	95
9.09	125	119	95

Table S-2. Comparison of the supercapacitor parameters of the PPy@$NiCo_2S_4$//rGO with previously published works.

Device	Binder	Capacitance	Energy density (Wh kg^{-1})	Power density (W kg^{-1})	Reference
$NiCo_2S_4$@PPy//AC	-	9.78 F cm^{-2}	34.62	120.19	1
$NiCo_2S_4$@$Ni(OH)_2$ @PPy//AC	-	9.11 F cm^{-2}	34.67	120.13	2
PPy@$NiCo_2S_4$ //N-CNTs	PTFE	908.1 F g^{-1}	50.82	160	3
PPy @$NiCo_2S_4$ NS//PPy @$NiCo_2S_4$ NS	PTFE	911 F g^{-1}	21.3	417	4
RGO/PPy/NiCo-LDH//RGO	PTFE	2534 F g^{-1}	41.9	698	5
PPy@$NiCo_2S_4$//rGO	-	2554.9 F g^{-1}	35.2	1472.3	This work

Table S-3. Details of fitting of experimental EIS results of different electrodes of $NiCo_2S_4$@PPy, $NiCo_2S_4$, and PPy/NF electrodes.

Rs (Ohm)	Rct (Ohm)	CPE Y_0 ($S.s^n$)	n1	Warburg Y_0 ($S.s^n$)	n2	Electrode
1.25	12.37	0.0051	0.71	0.014	0.5	PPy/NF
1.84	5.32	0.0042	0.82	0.015	0.62	$NiCo_2S_4$/NF
1.12	1.64	0.0099	0.66	0.011	0.61	$NiCo_2S_4$@PPy/NF

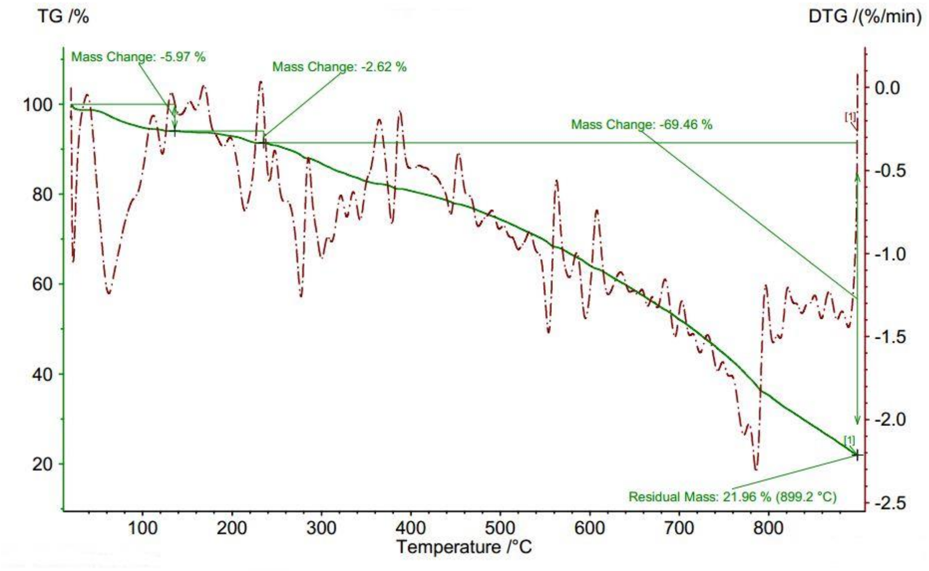

Figure S-1. Illustration of TG and DTG curves of pure PPy. The thermograms were recorded in the range of 25 to 900 °C under N_2 environment.

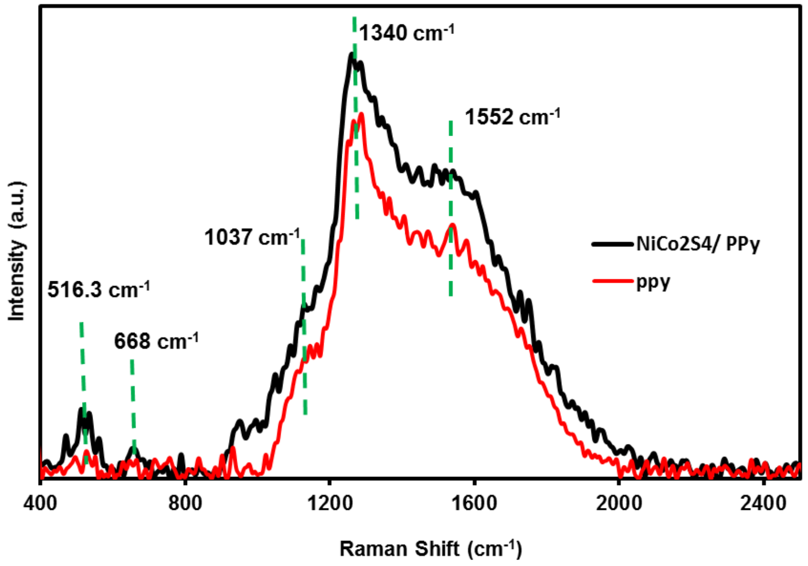

Figure S-2. Raman spectrum recorded for PPy and NiCo2S4/ PPy nanomaterials fabricated in the current work.

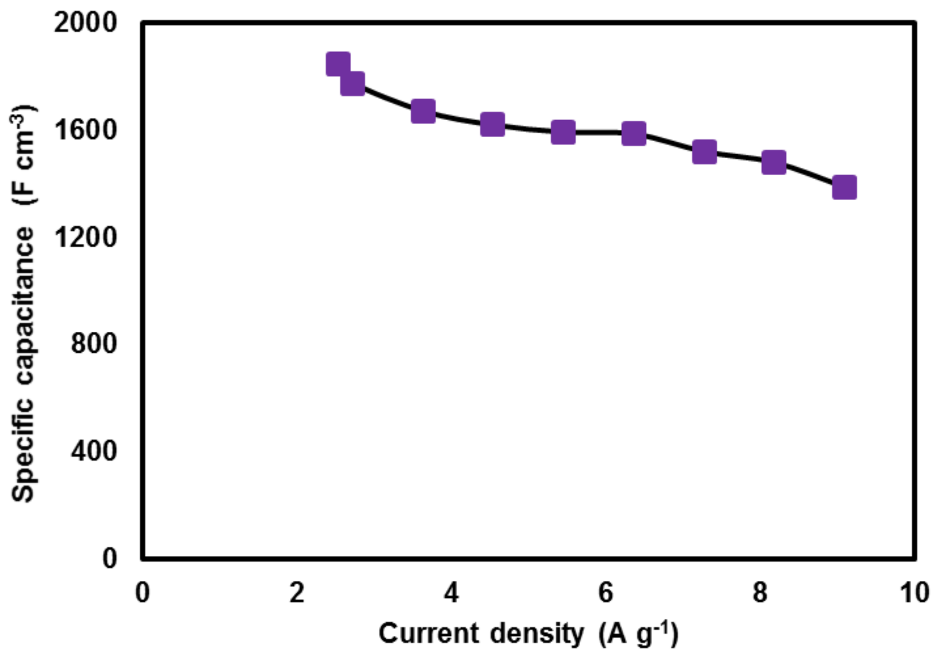

Figure S-3. diagram of calculated volumetric capacitance for $NiCo_2S_4$/ PPy.

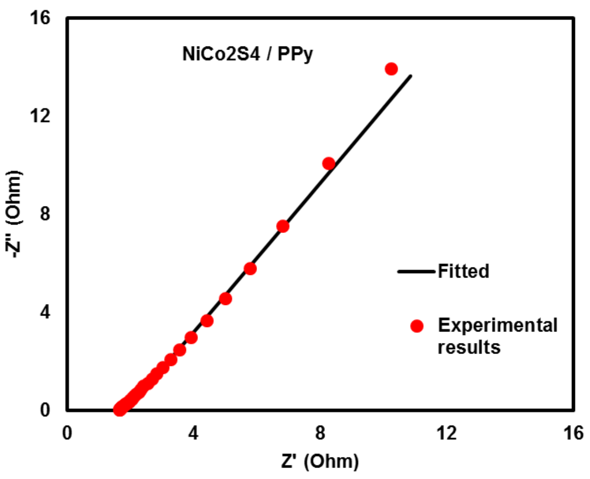

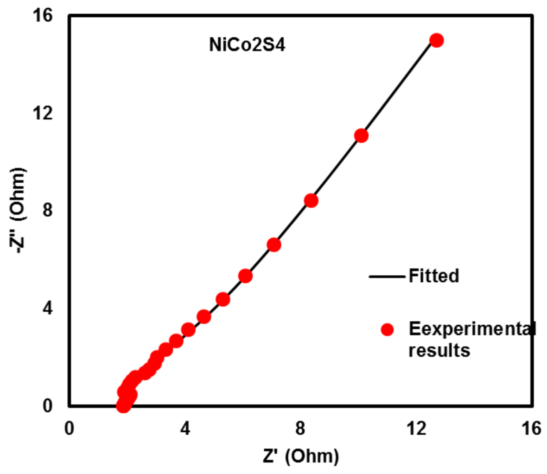

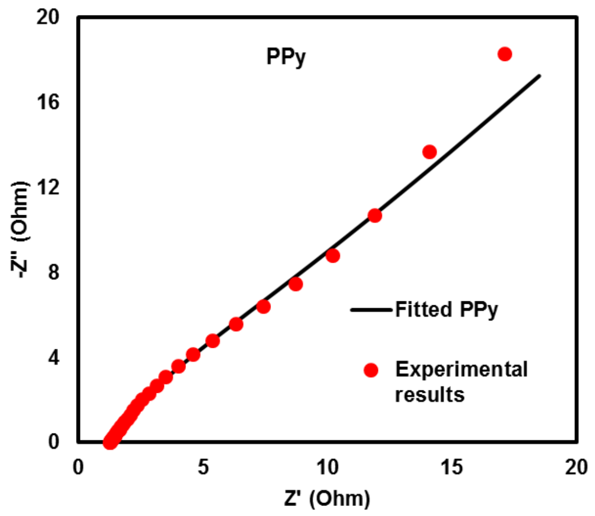

Figure S-4. Nyquist diagrams of each component before and after fitting.

References

1 Yan, M. *et al.* Construction of a Hierarchical NiCo2S4@PPy Core–Shell Heterostructure Nanotube Array on Ni Foam for a High-Performance Asymmetric Supercapacitor. *ACS Applied Materials & Interfaces* **8**, 24525-24535, doi:10.1021/acsami.6b05618 (2016).

2 Liang, M., Zhao, M., Wang, H., Shen, J. & Song, X. Enhanced cycling stability of hierarchical NiCo2S4@Ni(OH)2@PPy core–shell nanotube arrays for aqueous asymmetric supercapacitors. *Journal of Materials Chemistry A* **6**, 2482-2493, doi:10.1039/C7TA10413H (2018).

3 Zheng, Y. *et al.* Decoration NiCo2S4 nanoflakes onto Ppy nanotubes as core-shell heterostructure material for high-performance asymmetric supercapacitor. *Chemical Engineering Journal* **333**, 111-121, doi:https://doi.org/10.1016/j.cej.2017.09.155 (2018).

4 Zhang, J., Guan, H., Liu, Y., Zhao, Y. & Zhang, B. Hierarchical polypyrrole nanotubes@NiCo2S4 nanosheets core-shell composites with improved electrochemical performance as supercapacitors. *Electrochimica Acta* **258**, 182-191, doi:https://doi.org/10.1016/j.electacta.2017.10.102 (2017).

5 Liang, J. *et al.* Spacing graphene and Ni-Co layered double hydroxides with polypyrrole for high-performance supercapacitors. *Journal of Materials Science & Technology* **55**, 190-197, doi:https://doi.org/10.1016/j.jmst.2019.10.030 (2020).
